# The reverse remodeling of the aorta in patients after renal transplantation - the value of aortic stiffness index: prospective echocardiographic study

**DOI:** 10.1186/s12882-017-0453-5

**Published:** 2017-01-23

**Authors:** Tomasz Zapolski, Jacek Furmaga, Andrzej Jaroszyński, Anna Wysocka, Sławomir Rudzki, Andrzej P. Wysokiński

**Affiliations:** 1 Department of Cardiology, Medical University of Lublin, ul. Jaczewskiego 8, 20-950 Lublin, Poland; 2 Department of General and Transplant Surgery and Nutritional Treatment, Medical University of Lublin, Lublin, Poland; 3 Department of Family Medicine, Medical University of Lublin, Lublin, Poland; 4 Department of Nephrology, Jan Kochanowski University in Kielce, Kielce, Poland; 5 Department of Family Medicine and Geriatrics, Jan Kochanowski University in Kielce, Kielce, Poland; 6 Internal Medicine in Nursing Department, Medical University of Lublin, Lublin, Poland

**Keywords:** End-stage renal disease, Renal transplantation, Stiffness of aorta, Aortic stiffness index

## Abstract

**Background:**

Atherosclerosis is regarded as a combination of two major separate diseases: atherosis and sclerosis. Sclerotic component depends on deterioration of elastic properties of the aortic wall and is called aortic stiffness. The most valuable, non-invasive method of aortic stiffness assessment is echocardiography, which allows to calculate the aortic stiffness index (ASI). ASI is an independent predictor of all-cause and cardiovascular mortality in different groups of patients. The main aim of study was the assessment of the aortic reverse remodeling in patients with end-stage renal disease (ESRD) after renal transplantation (RT).

**Methods:**

Study group involved 42 patients aged 43.3 ± 12.6 years, including 19 women aged 49.9 ± 10.9 years and 23 men aged 41.5 ± 12.91 years, who have undergone RT from non-related renal transplant donors, The study protocol has been consisted of 5 stages: 1 week after RT, 3 months after RT, 6 months after RT, 1 year after RT and 3 years after RT. The echocardiographic examination was performed and measurements of Ao_max_, Ao_min_ were done. On the base of obtained parameters ASI, aortic distensibility (AD) and aortic strain (AS) were calculated according to adequate formulas.

**Results:**

The improvement of indices characterizing the elastic properties of aorta were noted. These changes attained the statistically significant level only at the end of the observation. ASI just after RT was equal – 4.65 ± 1.58, three months after RT – 4.54 ± 1.49, six months after RT – 4.59 ± 1.61, one year after RT – 4.35 ± 1.21 and three years after RT – 3.35 ± 1.29, while AD reached respectively – 6.55 ± 3.76 cm^2^/dyn^−1^10^−6^ just after RT, − 6.38 ± 3.42 cm^2^/dyn^−1^10^−6^ three months after RT, − 6.53 ± 3.60 cm^2^/dyn^−1^10^−6^ six months after RT, − 6.48 ± 2.79 cm^2^/dyn^−1^10^−6^ one year after RT and – 8.03 ± 3.95 cm^2^/dyn^−1^10^−6^ three years after RT. Noted AS values were equal – 6.61 ± 4.05%, just after RT, − 6.40 ± 3.58% three months after RT, − 6.56 ± 3.76%, six months after RT, − 6.45 ± 2.80% one year after RT, − 8.01 ± 3.97%. and three years after RT. The exact analysis of parameters concerning aortic function showed that to achieve ASI, AD and AS improvement, long time was needed, because the most significant changes of these indices were observed only between 1 year and 3 years after RT.

**Conclusions:**

There is a relationship between renal transplantation and improvement of the aortic elastic properties. The recovery of the renal function allows to initiate the reparative processes leading to at least partial restitution of the structure and features of the aorta, which is called reverse remodelling. Improvement of aortic wall elastic properties after renal transplantation is a continuous and prolonged process.

## Background

According to Windkessel’s concept [1] arterial system is the vascular network, which allows to modify an intermittent stream of blood outflowing from the heart to a continuous and stable blood flow through smaller vessels and capillaries. Among the many factors affecting the proper functioning of the system, one of the most important seems to be the compliance of the vessel wall, which is able to adapt (flow-dependent changes in diameter, cross sectional area of vessels) in response to changes in the pressure of blood flow [2]. A general term describing the resistance to deformation of the vessel under the influence of blood pressure is stiffness [3, 4].

Number of factors plays the role in the pathogenesis of aortic stiffness. Both physiological ones, which are especially important from a clinical point of view, as well as pathological, resulting from diseases directly or indirectly affecting the cardiovascular system, should be taken into consideration. It has been shown that the elastic properties of the aorta deteriorate with age. Except the age, also sex plays important role, because the process of stiffening of the aorta in women occurs about 10 years later than in men [5, 6]. The aging of the body is a crucial factor leading to reduced elasticity as a result of changes in the structure of collagen and elastin. However, congenital abnormalities of these proteins cannot be excluded, too. The study of Madeley et al. [7] indicates the important role of fibrillin-1 genotype in the pathogenesis of the aortic stiffness.

From the pathophysiological point of view, a common cause of aortic stiffness is undoubtedly atherosclerosis. Furthermore, atherosclerosis is now considered a combination of two major separate diseases: atherosis and sclerosis. Atherosis, developing as a result of the formation of atherosclerotic plaques, is a complicated process, in which inflammation plays a key role and one of the main and most common consequences of this condition is calcification of the plaque. The second element forming part of atherogenesis development is the deterioration of the large arteries elastic properties, which is defined as stiffness. Also crucial importance of metabolic factors like diabetes or end-stage renal disease (ESRD) should be mentioned [8].

In patients with ESRD aortic stiffness is increased [9]. Also renal function was described as a determinant of arterial stiffness despite of presence or absence of concomitant coronary artery disease [10]. In histological evaluation of arteries obtained from uraemic patients, some signs of fibroelastic intimal thickening, elastic lamellae calcification, and ground substance deposition were shown [11]. Regardless of the described morphological changes, there was proved, that in patients with ESRD, vascular endothelial function associated with the accumulation in the plasma of natural nitric oxide synthase (NOS) inhibitors, such as asymmetric dimethylarginine **(**ADMA) is seriously impaired [12]. These data suggest that ADMA seems to be a potential mediator linking the uremic milieu with the endothelial dysfunction underlying the increased arterial stiffness in patients with ESRD. Indeed ADMA as the endogenous NOS inhibitor accumulates in time of renal function deterioration [13].

Aortic stiffness affects the function of conducting through the aorta elevated blood pressure and changes the pressure curve, thus increasing the left ventricle (LV) afterload. This causes left ventricle hypertrophy (LVH) and also adversely modifies its diastolic and systolic function. Thickened LV becomes particularly sensitive to ischemia and both of these abnormalities increase the heterogeneity of the electrical activity of the heart [14].

### Aim of the study

The possibility of removal of the factor promoting the phenomenon of the vessel wall stiffening seems to be the most interesting non-pharmacological intervention. Considering the complex pathogenesis of aortic stiffness and limitations allowing only to modify the causative factors, mentioned action is purely hypothetical. The reasonable model of this situation represents a renal transplantation (RT), which due to curing the patient of one of the potential causes of aortic stiffness, gives hope to discover, as yet unexplored, modified elastic properties of the aorta.

The main objective of the study was, therefore, to evaluate the phenomenon of reverse remodeling of the aorta in patients with ESRD after RT.

## Methods

**Fig. 1:**
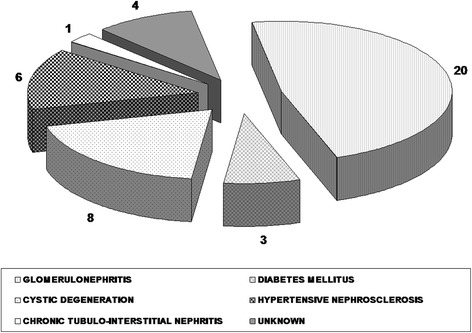
The etiology of ESRD in patients after RT

This is a small-scale progressive study using current echocardiographic methodology. Forty two patients, aged 43.3 ± 12.6 years, including 19 women aged 49.9 ± 10.9 years and 23 men aged 41.5 ± 12.91 years were enrolled to the study group. All patients have undergone RT from unrelated donors because of ESRD. In every patient examined in the study, the arteriovenous fistula was closed. Reasons for ESRD in the study group included: glomerulonephritis, diabetes mellitus, polycystic kidney degeneration, hypertensive nephrosclerosis, chronic tubulointerstitial nephritis and other unspecified causes (Fig. 1). Exclusion criteria were: heart failure, manifest coronary artery disease, patent arterio-venous fistula and unstable graft function at any time during the follow-up. To ensure that only ESRD related causes of aortic dysfunction were assessed, patients with mild to severe aortic valve disease on echocardiography were excluded from the study. All patients were treated with the immunosuppresion regimen, which included methylprednisolone, mycophenolate mofetil and cyclosporine microemulsion or tacrolimus (Table 1). Treatment prior to RT are shown in Table 1.

**Table 1:** Baseline characteristics of the study population

Characteristic	Value
age [years]	43.3 (±12.6)
gender (men/women) [n (%)]	23 (54.8%)/19 (43.2%)
time on dialysis until RT [months]	36.18 (±22.72)
haemoglobin [g/dl]	12.25 (±3.67)
natrium [mmol/l]	139.0 (±2.71)
potassium [mmol/l]	4.7 (±1.31)
magnesium [mmol/l]	0.99 (±0.28)
calcium [mmol/l]	2.54 (±0.24)
phosphorus [mmol/l]	1.68 (±0.49)
Ca X P [mg^2^/dl^2^]	52.47 (±13.94)
parathormone [pg/ml]	734.3 (±656.8)
creatinine [μmol/l]	539.24 (±245.75)
eGRF [ml/min/1,73 m^2^]	10.9 (±7.68)
urea [mmol/l]	9.74 (±5.72)
total protein [g/l]	75.0 (±14.3)
albumin [g/l]	4.6 (±1.13)
hs-CRP [mg/l]	2.12 (±2.22)
total cholesterol [mg/dl]	219.0 (±67.2)
LDL-cholesterol [mg/dl]	131.0 (±44.15)
HDL-cholesterol [mg/dl]	66.1 (±24.55)
triglycerides [mg/dl]	214.3 (±67.64)
glucose [mg/dl]	90.77 (±47.19)
troponin T [μg/l]	0.018 (±0.037)
myoglobin [ng/ml]	183.5 (±171.1)
creatine kinase [U/l]	81.1 (±86.2)
creatine kinase myocardial bound [U/l]	17.4 (±8.12)
treatment prior to RT	
ACE-inhibitors/sartans [n (%)]	0 (%)
calcium blockers [n (%)]	40 (95.2%)
beta-blockers [n (%)]	23 (54.8%)
alfa-blockers [n (%)]	5 (11.9%)
clonidine [n (%)]	31 (73.8%)
statins [n (%)]	3 (7.1%)
immunosuppression	
methylprednisolone [n (%)]	42 (100%)
mycophenolate mofetil [n (%)]	42 (100%)
cyclosporine [n (%)]	21 (50%)
tacrolimus [n (%)]	21 (50%)

In all patients a detailed medical history and physical examination were carried out. The following clinical parameters were evaluated: age, sex, duration of dialysis treatment to RT.

The study protocol consisted of 5 stages:

- Phase I - evaluation about 1 week after RT.
- Phase II - evaluation about 3 months after RT.
- Phase III - evaluation about 6 months after RT.
- Phase IV - evaluation about 1 year after RT.
- Phase V - evaluation about 3 years after RT.

During the subsequent phases the following measurements were performed:

Phase I:

- anthropometric measurements: height, weight. On the basis of these measurements the following indicators: body surface area (BSA), body mass index (BMI) have been calculated, using the appropriate formulas,
- measurements of pressure: systolic blood pressure (SBP), diastolic blood pressure (DBP). On the basis of these measurements mean blood pressure (MBP) and puls pressure (PP) have been calculated, using the appropriate formulas,
- heart rate (HR) measurement,
- transthoracic echocardiography (TTE) using the probe 2.5–3.5 MHz cooperating with echocardiography ultrasound system Sonos 5500 (Philips, Andover, MA, USA) and Sonos 7500 (Philips, Andover, MA, USA) was performed. In order to objectify the obtained data, the examination was conducted without prior detailed knowledge of the evaluated patient’s clinical data. All echocardiographic measurements were carried out according to the recommendations of the American and the European Society of Echocardiography [15].
- M-mode echocardiography has been performed under the control of two-dimensional imaging in the parasternal longitudinal axis. Aortic diameters were measured perpendicular to its long axis by TTE. In order to evaluate the parameters relating to the ascending aorta, ultrasonic beam was located 3 cm above the site of the aortic valve leaflets coaptation at the proximal tubular portion of the ascending aorta. Echocardiographic measurements were made using leading edge to leading edge method [16, 17]. Valid values in healthy subjects assessed by this method are as follows: at the end-diastole–28.5 ± 3.8 mm; at the mid-systole - 30.0 ± 3.6 mm [18]. The following parameters were recorded:
- aortic maximal diameter - Ao_max,_ [mm] – corresponding to the systolic dimension of the aorta; was measured at the time of full opening of the aortic valve leaflets,
- aortic minimal diameter - Ao_min_, [mm] – corresponding to the diastolic dimensions of the aorta; was measured on the peak of QRS wave of simultaneously recorded electrocardiogram.
- interventricular septum systolic diameter - IVSSd, [mm],
- interventricular septum diastolic diameter - IVSDd, [mm],
- posterior wall systolic diameter - PWSd, [mm],
- posterior wall diastolic diameter – PWDd, [mm],
- left ventricle endsystolic diameter LVESd, [mm],
- left ventricle enddiastolic diameter – LVEDd, [mm].

Then, on the basis of the above listed planimetric measurements the functional indices of the aorta have been calculated [3, 19]:

- aortic strain – AS, [%]; AS = (Ao_max_ – Ao_min_)/Ao_min_,
- aortic distensibility – AD, [cm^2^/dyn^−1^10^−6^]; AD = [2 x (Ao_max_ – Ao_min_)]/[(Ao_min_ x PP)]; PP was the difference in SBP and DBP measured at the right brachial artery simultaneously with measuring the dimensions of the aorta [17, 20],
- aortic stiffness index – ASI, [n]; ASI = log {[SBP/DBP/(Ao_max_ – Ao_min_)]/Ao_min_}; SBP and DBP were measured on the right brachial artery simultaneously with assessing the dimensions of the aorta.

Based on planimetric parameters, indices of LV structure and function have been calculated as follows [21, 22]:

- left ventricle endsystolic volume – LVESV, [ml] - calculated according to the Teichholz formula: LVESV = [7/(2,4 + LVESd)] x [LVESd]^3^,
- left ventricle enddiastolic volume - LVEDV, [ml] - calculated according to the Teichholz formula: LVEDV = [7/(2,4 + LVEDd)] x [LVEDd]^3^,
- left ventricle stroke volume – SV, [ml] - calculated according to the formula: SV = LVEDV – LVESV,
- stroke index – SI, [ml/m^2^] - calculated according to the formula: SI = SV/BSA,
- cardiac output – CO, [l/min] - calculated according to the formula: CO = SV x HR,
- cardiac index – CI, [l/min/m^2^] - calculated according to the formula: CI = CO/BSA,
- left ventricle ejection fraction – EF, [%] - calculated according to the formula: EF = [(LVEDV – LVESV)/LVEDV] x 100,
- left ventricle shortening fraction – FS, [%] - calculated according to the formula: FS = [(LVEDd – LVESd)/LVEDd] x 100,
- left ventricle mass – LVM, [g] - calculated according to the formula [23]: LVM = 1,04 x [(IVSDd + PWDd + LVEDd)^3^] - 13,6 g
- left ventricle mass index – LVMI, [g/m^2^] - calculated according to the formula: LVMI = LVM/BSA.
- left ventricle hypertrophy - LVH was recognized if [24]:
- LVMI > 131 g/m^2^ in men,
- LVMI > 100 g/m^2^ in women.

### Statistical analysis

The data were statistically analyzed using the Statistica 6.0 software (StatSoft Inc.). Quantitative and qualitative data of the study subgroups and clinical variables were analyzed descriptively. The number of patients in particular groups in statistical analyzes in the tables was marked with the symbol “n”. Differences between quantitative variables relating to the normal distribution were analyzed using Student’s *t* test for dependent variables. In order to compare several variables the analysis of variance (ANOVA) for the dependent and independent variables was used. In the case of dependent variables (further measurements in patients after RT) analysis of Friedman variance ranks (Friedman ANOVA) was used and Kendall’s coefficient of concordance was provided. The comparative analysis of multiple dependent variables was additionally calculated by ANOVA test with repeated measurements. In order to determine the relationship between two variables the Spearman rank correlation coefficient was used.

*P* value 0.05 was assumed as the level of significance of statistical tests. In the case of *p-*value less than 0.00001, 0.0001, or 0.001 its value was presented as a significance level of *p <*0.00001, *p <*0.0001 and *p <*0.001, respectively. In other situations, the exact result was given.

## Results

### Characteristics of the study group

The study group consisted of 42 patients including 19 women and 23 men after RT due to ESRD. Table 1 shows the baseline characteristics of the study population.

A detailed analysis of changes in HR, pressures and anthropometric data are presented in Table 2. HR during the observation gradually decreases and after a period of 3 years was significantly lower than at the baseline (Table 2). Systolic blood pressure in the course of the follow-up steadily significantly declined, while diastolic blood pressure remained unchanged (Table 2). As a result, the PP reduction was statistically significant (Table 2).

**Table 2:** Blood pressure, HR and BMI in patients after RT

Parameter	immediately after RT	3 months after RT	6 months after RT	1 year after RT	3 years after RT	*P*						
	1 (*n =* 42)	2 (*n =* 42)	3 (*n =* 42)	4 (*n =* 42)	5 (*n =* 42)	1vs2	1vs3	1vs4	1vs5	2v3	3vs5	4vs5
HR [n/min]	76.97 (±8.86)	75.4 (±7.82)	75.8 (±8.32)	73.2 (±7.97)	72.9 (±37.46)	0.453	0.247	0.123	0.034	0.875	0.082	0.142
SBP [mmHg]	138.8 (±13.0)	135.8 (±12.8)	135.3 (±11.4)	134.6 (±14.5)	133.6 (±10.5)	0.023	0.027	0.009	<0.001	0.659	0.219	0.342
DBP [mmHg]	87.7 (±8.2)	86.3 (±10.2)	86.8 (±8.7)	87.6 (±8.4)	88.3 (±7.4)	0.723	0.572	0.745	0.561	0.435	0.563	0.843
PP [mmHg]	51.0 (±9.1)	50.2 (±8.2)	49.3 (±9.1)	46.8 (±8.2)	45.3 (±6.0)	0.356	0.281	0.002	<0.001	0.265	0.017	0.453
BSA [m^2^]	1.87 (±0.12)	1.87 (±0.12)	1.87 (±0.12)	1.86 (±0.13)	1.86 (±0.13)	NA	NA	NA	NA	NA	NA	NA
BMI [kg/m^2^]	24.05 (±3.78)	24.05 (±3.78)	24.05 (±3.78)	24.11 (±3.54)	24.21 (±3.67)	NA	NA	NA	NA	NA	NA	NA

*NA* not applicable

Progressive reduction of evaluated planimetric dimensions of the LV was noted. At the end of the follow-up, all planimetric indices were significantly lower than immediately after surgery. The dynamics of these changes, however, was different depending on assesed parameters in subsequent periods of observation (Table 3). This was accompanied by the change of evaluated LVH indices. After the 3-years follow-up planimetric indices were considerably lower in comparison with the baseline, but the statistical significance of these reductions was proved only after one year follow-up (Table 3). As a result of the planimetric parameters significant decline, the volumetric parameters of LV decrease was observed. Their values at the end of the observation became significantly lower than at the beginning, but the dynamics of the particular parameters behaved very individually (Table 3). Regarding the indices of LV contractility significant changes in EF and FS were observed (Table 3).

**Table 3:** Planimetric, mass, volumetric and hemodynamic parameters of the LV and the parameters of LVH in patients after RT

Parameter	immediately after RT	3 months after RT	6 months after RT	1 year after RT	3 years after RT	*P*						
	1 (*n =* 42)	2 (*n =* 42)	3 (*n =* 42)	4 (*n =* 42)	5 (*n =* 42)	1vs2	1vs3	1vs4	1vs5	2v3	3vs5	4vs5
LV dimensions												
LVEDd [mm]	52.5 (±5.1)	50.7 (±4.5)	50.5 (±4.6)	50.1 (±4.7)	49.7 (±4.8)	0.047	0.017	0.001	<0.001	0.953	0.072	0.247
LVESd [mm]	35.1 (±5.5)	34.6 (±3.7)	34.4 (±4.3)	33.9 (±4.9)	32.0 (±4.7)	0.153	0.141	0.011	0.004	0.580	0.007	0.009
PWDd [mm]	12.4 (±1.4)	12.2 (±1.6)	11.9 (±1.3)	11.8 (±1.7)	11.2 (±1.2)	0.91	0.01	0.233	<0.001	0.684	0.001	0.072
PWSd [mm]	15.3 (±1.2)	16.3 (±1.5)	16.6 (±2.2)	16.0 (±2.3)	17.4 (±2.0)	0.125	0.007	0.580	<0.001	0.450	0.001	0.027
IVSDd [mm]	13.4 (±1.4)	13.3 (±0.9)	13.0 (±0.9)	12.1 (±0.8)	11.8 (±1.3)	0.366	0.363	0.003	<0.001	0.142	0.01	0.03
IVSSd [mm]	16.1 (±1.5)	16.3 (±1.3)	17.6 (±2.0)	16.8 (±2.0)	17.3 (±1.9)	0.680	<0.001	0.034	<0.001	0.175	0.523	0.980
LVM indices												
LVM [g]	329.5 (±73.4)	330.1 (±77.2)	323.0 (±74.9)	309.9 (±74.9)	283.9 (±62.0)	0.857	0.254	0.008	<0.001	0.189	<0.001	0.007
LVMI [g/m^2^]	173.9 (±38.6)	173.4 (±34.9)	171.2 (±35.6)	165.3 (±34.2)	149.3 (±33.0)	0.784	0.243	0.011	<0.001	0.124	<0.001	0.004
LV volumetric parameters												
LVEDV [ml]	134.5 (±29.2)	124.2 (±31.3)	123.3 (±28.9)	122.2 (±34.5)	118.5 (±25.7)	0.012	0.009	0.002	0.001	0.834	0.052	0.274
LVESV [m]	53.25 (±19.90)	50.13 (±13.56)	50.43 (±15.61)	48.56 (±18.52)	42.35 (±15.34)	0.146	0.118	0.021	0.005	0.439	0.008	0.011
SV [ml]	81.31 (±19.36)	78.9 (±15.60)	77.8 (±17.62)	76.5 (±21.1)	76.23 (±18.05)	0.033	0.028	0.023	0.013	0.486	0.754	0.765
Cardiac output parameters												
CO [l/min]	6.16 (±0.76)	5.81 (±0.59)	5.77 (±0.71)	5.54 (±0.82)	5.55 (±0.68)	0.019	0.014	0.011	0.007	0.685	0.232	0.983
CI [l/min/m^2^]	3.29 (±0.49)	3.10 (±0.47)	3.09 (±0.51)	2.97 (±0.45)	2.98 (±0.51)	0.021	0.017	0.013	0.009	0.832	0.274	0.934
Contractility parameters												
EF [%]	60.98 (±9.93)	59.25 (±8.92)	60.54 (±9.34)	61.03 (±10.11)	64.59 (±9.11)	0.865	0.659	0.184	0.008	0.236	0.009	0.018
FS [%]	33.36 (±7.01)	32.67 (±6.72)	33.02 (±7.34)	33.78 (±9.32)	35.83 (±6.58)	0.753	0.769	0.238	0.011	0.327	0.012	0.045

Comparative analysis of the ascending aorta dimensions revealed a significant increase in the Ao_max_ after 3 years follow-up. This change, however, was statistically significant only in the final phase of the follow-up. However Ao_min_ during the study remains unchanged (Table 4). As a result of the changes of Ao_max_ and accompanied, described above, changes in the value of pressure, a noticeable improvement of indicators characterizing the stiffness of the aorta was observed. These changes, however, reached statistical significance only at the end of the follow-up (Table 4).

**Table 4:** Planimetric dimensions and functional indicators of the aorta in patients after RT

Parameter	immediately after RT *n =* 42	3 years after RT *n =* 42	*P*
Ao_max_ [mm]	33.17 (±4.06)	33.81 (±3.89)	0.049
Ao_min_ [mm]	31.24 (±4.58)	31.41 (±4.34)	0.594
ASI [n]	4.65 (±1.58)	3.35 (±1.29)	<0.001
AD [cm^2^/dyn^−1^10^−6^]	6.55 (±3.76)	8.03 (±3.95)	<0.001
AS [%]	6.61 (±4.05)	8.01 (±3.97)	<0.001

### Analysis of changes in echocardiographic parameters concerning the aorta during the 3-years follow-up

**Fig. 2:**
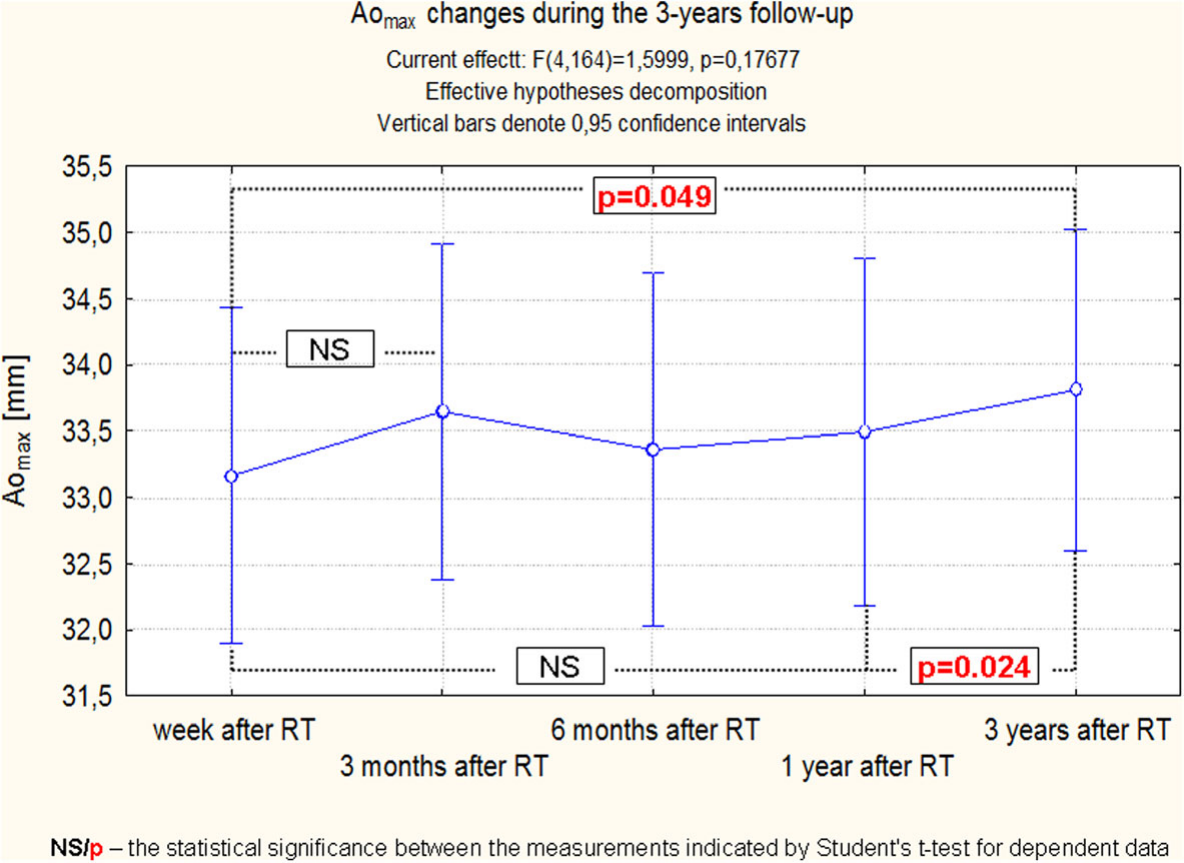
Changes Ao_max_ during the 3-year follow-up of patients after RT. Trend change for the entire period of observation - test ANOVA with repeated measurements and comparison of measurements in different periods of observation – Student’s *t* test for dependent variables

**Fig. 3:**
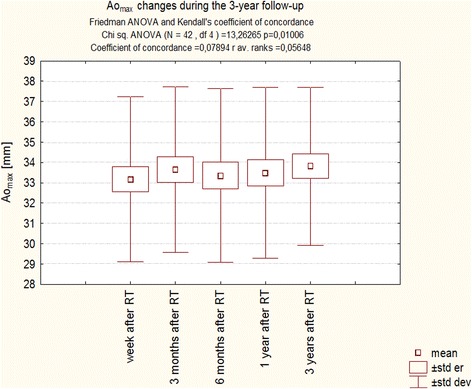
Changes Ao_max_ during the 3-year follow-up of patients after RT. Anova Friedman and Kendall’s coefficient of concordance

A detailed analysis of Ao_max_ changes indicates that differences concerning this parameter are extremely subtle. Despite the significant changes found in the Student’s *t* test indicating a statistically significant increase of this dimension, dependent mainly on changes in the final phase of the follow-up, simultaneous analysis of all measurements in the test ANOVA with repeated measurements did not reveal the significance of changes (Fig. 2). In contrast, the same analysis performed using Friedman Anova method confirmed the statistical significance (Fig. 3).

**Fig. 4:**
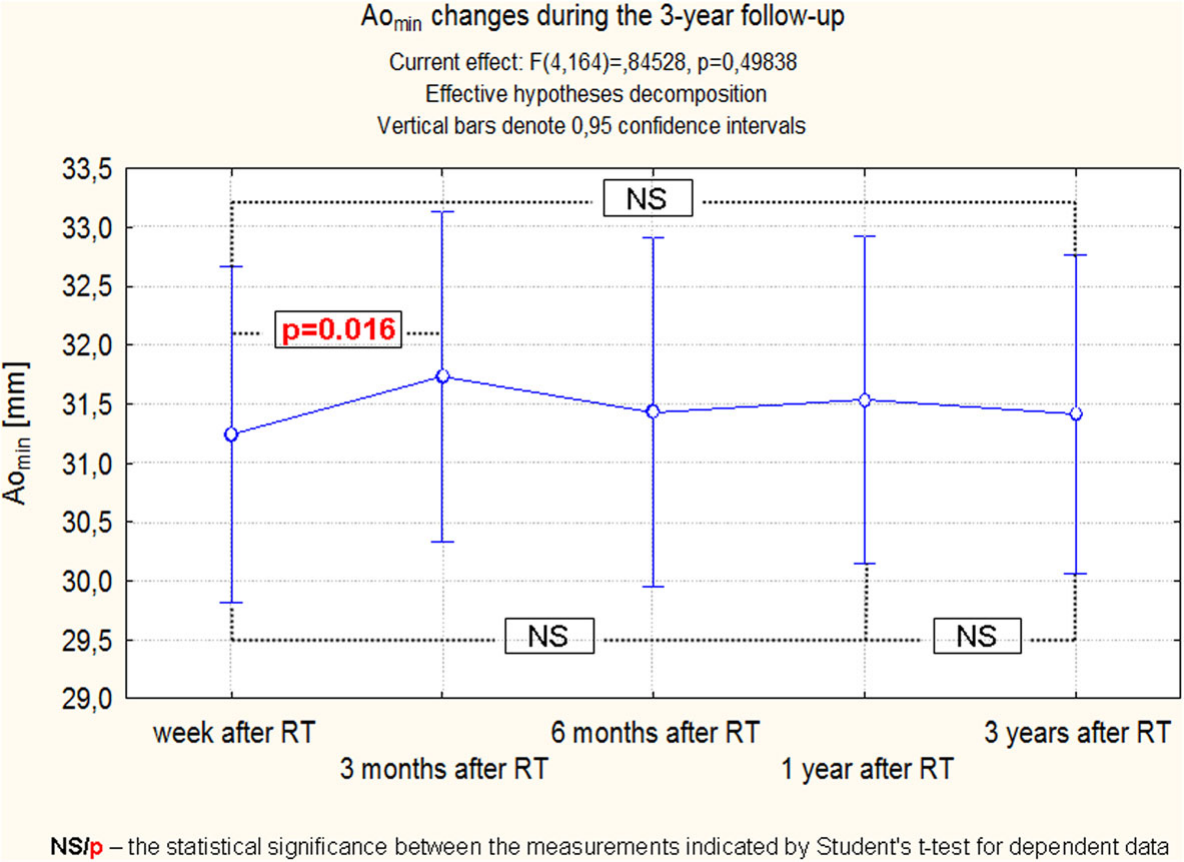
Changes Ao_min_ during the 3-year follow-up of patients after RT. Trend change for the entire period of observation - test ANOVA with repeated measurements and comparison of measurements in different periods of observation – Student’s *t* test for dependent variables

**Fig. 5:**
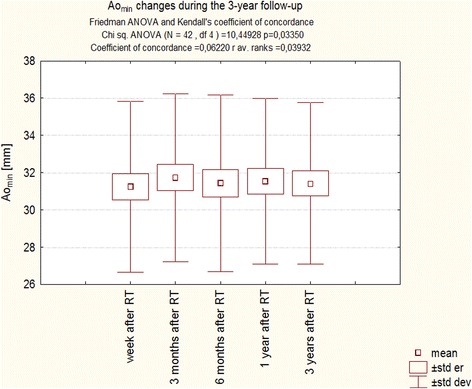
Changes of Ao_min_ during the 3-year follow-up of patients after RT. Anova Friedman and Kendall’s coefficient of concordance

Analogously, Ao_min_, evaluated by ANOVA with repeated measures did not change significantly over the 3-years follow-up (Fig. 4), but assessed using Friedman Anova slightly but significantly decreased (Fig. 5).

**Fig. 6:**
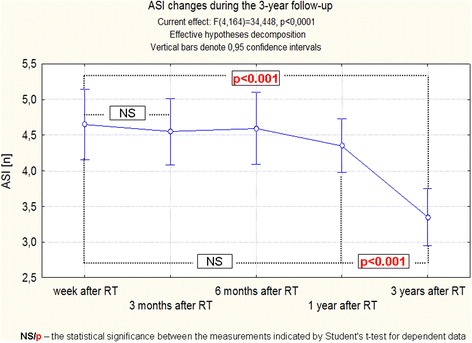
Changes of ASI during the 3-years follow-up of patients after RT. Trend change for the entire period of observation - test ANOVA with repeated measurements and comparison of measurements in different periods of observation – Student’s *t* test for dependent variables

**Fig. 7:**
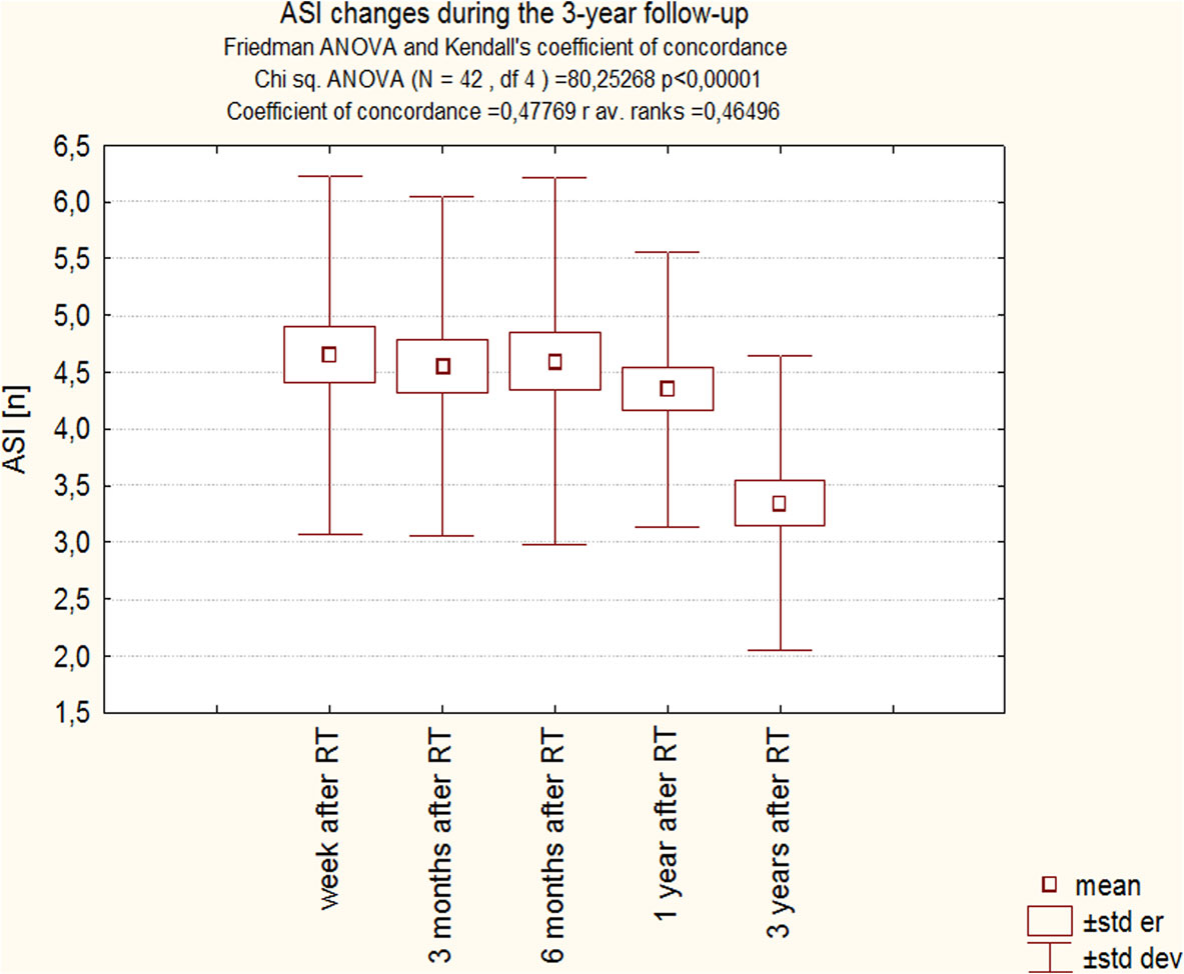
Changes of ASI during the 3-years follow-up of patients after RT. Anova Friedman and Kendall’s coefficient of concordance

**Fig. 8:**
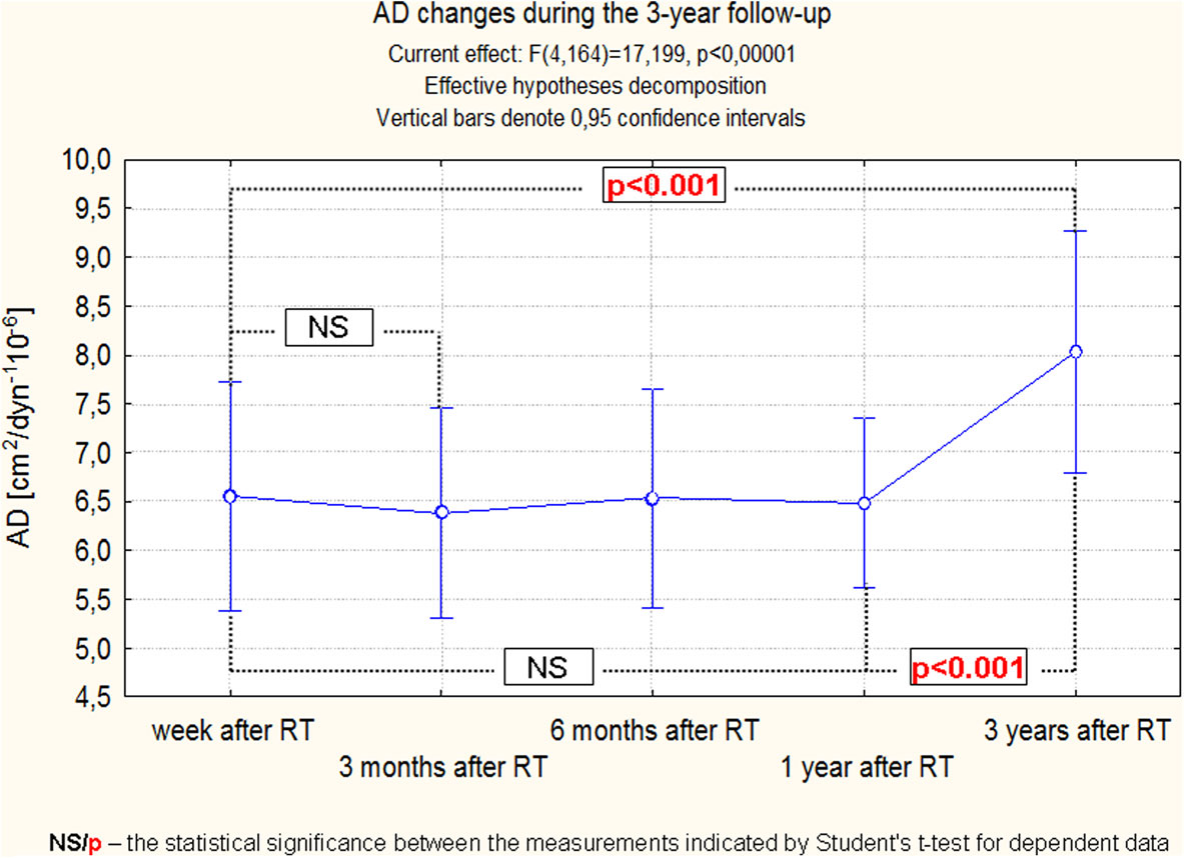
Changes of AD during the 3-year follow-up of patients after RT. Trend change for the entire period of observation - test ANOVA with repeated measurements and comparison of measurements in different periods of observation – Student’s *t* test for dependent variables

**Fig. 9:**
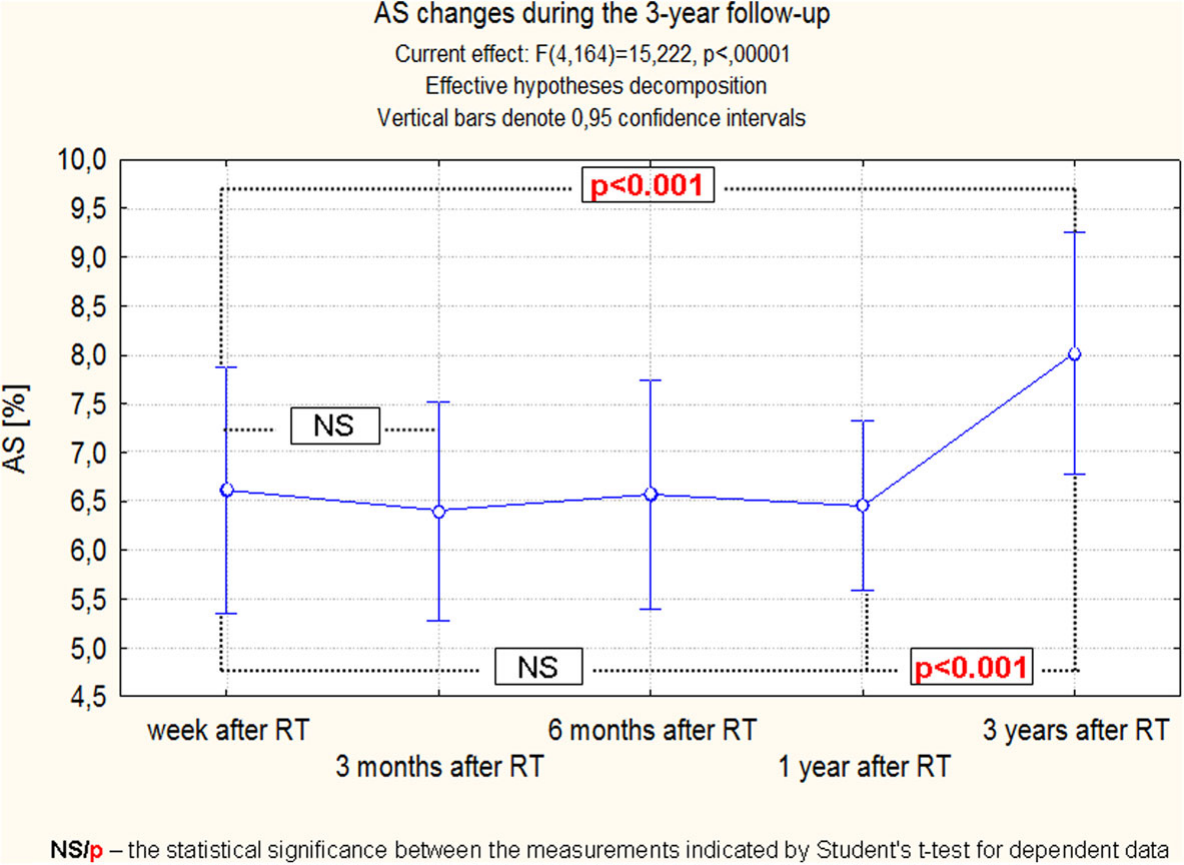
Changes of AS during the 3-year follow-up of patients after RT. Trend change for the entire period of observation - test ANOVA with repeated measurements and comparison of measurements in different periods of observation – Student’s *t* test for dependent variables

These small changes of the aorta dimensions and the previously discussed changes of pressure, caused that differences in the functional indices of the aorta were highly significant, regardless of the method of statistical analysis. Moreover, detailed analysis showed that changes of ASI, AD and AS require a long time to reveal, because the most significant differences between the observations were noticed between a year and three years after RT (Figs. 6, 7, 8, 9).

### Analysis of the relationship between ASI and the selected echocardiographic parameters

Using the Spearman’s rank correlation a detailed analysis of ASI with the tested echocardiographic parameters was carried out. Among the many echocardiographic indices obtained just after RT significant correlation with ASI and LVESd, PWSd, IVSSd, LVESV, LVM and EF was observed (Table 5). The analysis of parameters obtained at the end of a 3-year follow-up showed a statistically significant relationship between LVESd, PWSd, LVEDV, LVESV, EF, FS and the ASI (Table 5).

**Table 5:** Correlation analysis ASI with echocardiographic parameters in patients immediately after RT and after the 3-year follow-up

Parameter	ASI			
	immediately after RT		after 3 years follow-up	
	R	P	R	P
LVEDd	0.145	0.358	0.037	0.813
LVESd	0.321	0.038	0.365	0.019
PWDd	0.143	0.365	0.037	0.813
PWST	0.321	0.038	0.346	0.026
IVSDd	0.126	0.425	0.103	0.513
IVSSd	0.313	0.043	0.085	0.590
LVEDV	0.003	0.980	0.349	0.014
LVESV	0.382	0.012	0.330	0.032
LVSV	−0.028	0.857	−0.089	0.572
LVM	0.308	0.046	0.096	0.545
LVMI	0.017	0.913	0.286	0.066
EF	−0.348	0.020	−0.367	0.027
FS	−0.280	0.072	−0.321	0.024
Ao_max_	−0.234	0.135	−0.223	0.213
Ao_min_	0.265	0.098	0.283	0.086
AD	−0.603	<0.0001	−0.588	<0.0001
AS	−0.619	<0.0001	−0.595	<0.0001

Correlation Spearman’s rank test

Multivariate analysis performed by multiple regression revealed that among the echocardiographic parameters analyzed immediately after RT, PWSd LVH and AD were independently associated with ASI (Table 6), but the assessment made after the 3-years follow-up showed that LVESd, PWSd, LVESV, FS and AS were independently associated with ASI (Table 7).

**Table 6:** Independent echocardiographic parameters related to ASI in patients immediately after RT

Dependent variable	Independent variable	β	B	st. dev.	*p*
ASI	PWSd	0.322	4.305	2.058	0.050
	LVM	−0.270	−0.006	0.003	0.050
	AD	−1.144	−0.462	0.171	0.014

Multiple linear regression analysis; model: R = 0,932, R^2^ = 0,870, *p =* 0,00002

**Table 7:** Independent echocardiographic parameters related to ASI in patients 3 years after RT

Dependent variable	Independent variable	β	B	st. dev.	*p*
ASI	LVESd	−1.004	−2.893	0.0003	0.00007
	PWSd	−0.165	−1.342	0.00009	0.00004
	LVESV	0.345	0.031	0.000009	0.0001
	FS	0.389	0.084	0.000020	0.0001
	AS	−0.503	−0.167	0.000003	0.00001

Multiple linear regression analysis; model: R = 1,0, R^2^ = 1,0, *p <* 0,00001

## Discussion

The conducted comparative assessment of the planimetric parameters of the LV showed a reduction of its dimensions during the 3-years follow-up, with a significant reduction of LVEDd, LVESd, LVEDV and LVESV noticeable already in the short term, after the first three months of observation. Similarly, the indices characterizing LVM also have been considerably reduced, but the time necessary for the existence of these changes was, as proven, much longer. The greatest reduction in LVM and LVMI was observed between measurement performed 1 year after RT and in the final phase of follow-up.

In the current study, during the 3-years after RT, LVMI decreased from 173.9 ± 38.6 g/m^2^ to 149.3 ± 33.0 g/m^2^. These results are similar to those outlined by Parffrey et al. [25], who found in patients after transplantation LVH reduction of this parameter from 166 g/m^2^ to 135 g/m^2^. Decreased LVM was accompanied by a reduction in its dimensions, that was reflected by a significant reduction LVEDV of LVEDV from 116 ml/m^2^ to 89 ml/m^2^. Montanaro et al. [26] in study conducted admittedly in a small, because consisting of 23 individuals, group of patients, demonstrated a reduction of LVMI from 161.4 g/m^2^ to 122.1 g/m^2^ after the 2-year follow-up. The recruitment to achieve the observed positive changes, however, is not only to perform the transplant, but also to maintain the adequate function of the transplanted organ. Indeed, Ferreira et al. [27] have shown that both, dimensions LV and LVM, decreased only in those patients after RT, in which the function of the transplanted organ was correct, what was manifested, on the one hand, as normalization of serum creatinine, and on the other as the normalization of blood pressure. In patients with poor-controlled mentioned above parameters, favorable reverse LV remodeling do not occur. Reduction of LVM, together with a reduction in planimetric and volumetric parameters of LV carries important prognostic information. This was proven by Sharma et al. [28], who underlined the fact, that LVESd and the maximum thickness of the LV wall, subsequent to the calcification of the mitral annulus, are independent predictors of survival after RT. They postulate to consider the recommendation of these echocardiographic parameters as standard prognostic factors used in assessing the risk of mortality in patients after RT.

Historically, LV systolic dysfunction was regarded as the limitation for the selection of patients appropriate for RT. That was probably the reason that observed in the study group parameters of LV systolic function were normal. However, initially normal EF and FS increased after 3 years and the improvement of these parameters, although seemingly little in absolute terms, was statistically significant. The data from the literature suggest that the greatest benefit of RT in relation to the LV systolic function refers to patients with very impaired contractility. Melchior et al. [29] investigated a group of 29 patients with low EF and showed an increase of the average EF from 37.8% to the value of almost normal, as much as 52% over the short, because only 1-month follow-up. Further observation showed further improvements in contractility, because after a year of follow-up EF was already 58.2%. Other studies conducted by Wali et al. [30] confirmed the beneficial effect of RT on LV systolic function. During the 6-month follow-up investigators showed an increase in EF from 31.6% to 47.2%, and over the next six months less dynamic growth of EF to 52.2% were noted. Improvement of contractile function was accompanied by a significant improvement in functional capacity expressed by a reduction in NYHA class. Other studies, as well as the presented research, indicate the growth of FS. Parfrey et al. [25] reported that RT is associated with an increase in FS from 21.5% to 33.4%. In all of these and most of other available studies the time of follow – up was relatively short and do not exceed a year of observation. In this context, it should be emphasized the importance of the current 3-years follow-up, revealing that in the long term, patients even with normal systolic function after RT could achieve further benefits of this form of treatment.

Indices of aortic function as ASI, AD and AS during the 3-year follow-up improved significantly. It may indicate, that changes occurring after RT allow for at least partial retrieve of the aorta elastic properties. Evaluation of the exact changes in the subsequent stages of the observation proves that the beneficial effect of the final transformation occurs between one year observation and the assessment made at the end of the follow-up after 3 years. This proves that the process of returning elastic properties of the aorta is a long-term phenomenon. Histopathological examination of the arteries performed in patients with ESRD indicates the presence of the process of remodeling, which consists of two main elements: atherosis and sclerosis [31, 32]. The coronary artery disease (CAD), as a form of arteriosclerosis, is particularly associated with increased aortic stiffness. Arteriosclerosis and atherosclerosis share similar pathobiologic processes [3]. Coronary artery calcification is associated with impaired aortic distensibility [33]. Arterial stiffness has long been viewed as a consequence of long-standing hypertension, which is the fixed component of kidney failure. However, recent studies have suggested that arterial stiffness may contribute to the pathogenesis of hypertension [34]. Other comorbidities have a large effect on aortic stiffness. Diabetes mellitus is independently associated with lower aortic distensibility, increased arterial stiffness and impaired flow-mediated dilatation [35]. Less common diseases like, aortic regurgitation, congenital heart disease (bicuspid aortic valve, tetralogy of Fallot), connective tissue disorders (Marfan syndrome, Ehlers-Danlos syndrome), aortic coarctation repair, hypertophic cardiomyopathy, can also cause impairment of aortic elastic properties [3].

From an epidemiological point of view, RT improves cardiovascular prognosis in this group of patients [36]. This improvement appears to be dependent on many factors, among which the recovery of normal glomerular filtration rate (GFR), enabling elimination of uremic toxins [37] as well as better control of cardiovascular risk factors [38] should be mentioned. The elastic properties of the aorta may be affected by a medication taken by patients. Mitchell et al. demonstrated, that long-term trandolapril treatment is associated with reduced aortic stiffness [39]. The angiotensin receptor blockers (sartans) may produce the same effect as ACE inhibitors. Treatment with ibersartan improved arterial compliance, whereas inflammatory and endothelial markers remained unchanged [40]. Additionally, it was proved, that in patients with chronic kidney disease a combination of ACE inhibitor and sartans allows to achieve even greater reduction of arterial stiffness than the treatment with only one of mentioned drugs [41]. Beta-blockers have different, often opposing action on the arterial elastic properties. Nebivolol, which is a selective beta-1 blocker with nitric oxide potentiating vasodilatatory effect, has been shown to slightly decrease the central pressure indices, when compared to atenolol [42]. The calcium channel blocker were evaluated among a large number of studies, where it proved to reduce central blood pressure and arterial stiffness [43]. In patients with CAD and normal blood pressure the treatment with statin provided significant decrease of PWV and improvement of arterial stiffness, what was not observed in patients with CAD and hypertension [44]. But we should not forget that the RT and the associated necessary drug therapy is connected with the new, previously absent factors, potentially adversely affecting the elastic properties of the aorta. Among them essential therapy with cyclosporine, corticosteroids, azathioprine and mycophenolate mofetil should be taken into consideration. These drugs, necessary to maintain the graft, cause hypertension, stimulate LVH and promote metabolic disorders as diabetes or hypercholesterolemia, potentially increasing the stiffness of the aorta.

Consequently, the altered elastic properties of the aorta represent the net, responsible for the disappearance of the old risk factors of aortic stiffness and the appearance and the potency of new ones. The evolution of aortic stiffness after RT is poorly understood. While much is already known about the relationship of ESRD and the pathogenesis of aortic stiffness and its prognostic significance, the issue of potential reverse remodeling of the aorta becomes a recent interest of researchers. Studies provided up to date, were based on the measurement of aorto-femoral pulse rate, which is an admitted method of assessing of arterial stiffness [45]. According to our knowledge, any study regarding aortic stiffness in patients after RT with using the direct serial assessment of aortic functions, as it was done in the present work, has not been carried out, yet. This method has been successfully validated in other clinical applications [3, 46].

Some authors have shown significant improvement in arterial elasticity [47], while in other studies, no improvement of the flexibility of vessels after RT has been observed [48]. Bachelet-Rousseau et al. [49] did not show a significant reduction in arterial stiffness after RT. Similarly, in the study provided by Zoungas et al. [48], evaluating 36 patients after RT, there were no improvement of elastic properties of the aorta, however, mentioned authors demonstrated a significant reduction of the femoral artery stiffness. All of these studies were provided in the relatively short, because the period of observation did not exceeding one year. In these conditions currently obtained data are even more valuable. Extending the time of patients monitoring, allows to notice, that a phenomenon of favorable remodeling of the aorta is possible, and to indicate that for the assessment of the available test methods quite a long time is required. In accordance with the results of the current study are the data presented by de Lima et al. [50]. In the group of 32 patients after RT followed for a relatively long time, lasting 40 months, cited above authors showed an improvement of indirect indices related to the stiffness of the aorta such as LVH and carotid arterial compliance and remodeling of the thickened at the baseline intima-media complex of the carotid artery. Although the present study indicate changes in the elastic properties of the arteries, beneficial in the long-term period, it cannot be expected to complete their restitution to the level observed in healthy subjects [51]. This depends in part on the irreversible changes, and partly on the appearance of previously mentioned, new immunosuppressive treatment-related factors, adversely modifying elastic properties of the aorta. The efficiency of the transplanted organ and the presence of subclinical inflammation features is also not without significance [51].

Aortic stiffness is also in direct connection with the rejection of the graft. In the study provided by Bahous et al. [52] it was demonstrated that the transplanted organ injury long time after the RT increases the stiffness of the aorta. This relationship occurs is a time-dependent menner and it does not depend on such factors as age, mean arterial pressure, atherosclerosis coexistence, drug therapy and even the presence of the recipient’s own kidneys. It is also not clear, whether these beneficial changes of the aorta elastic properties are at least partially dependent on decreasing, as far as the observation period extends, blood pressure. In the present study, a decrease in systolic blood pressure was observed over almost all of the observation periods. In contrast, the value of the pulse pressure evaluated at the beginning of the study, was significantly reduced after a year of the observation. Taking into consideration that the method of the calculation of the aorta elastic properties indicators includes both, the size of the aorta as well as blood pressure and pulse pressure, it can not to be excluded that the observed changes are not solely dependent on the regression changes or reverse remodeling of the aortic wall. On the other hand, it is well known, that the stiffness of the aorta and blood pressure are in mutual feedback [53]. This relationship concerns, on the one hand, the pathogenic mechanisms, resulting from elevated blood pressure, which stimulate the stiffening of the aorta. On the other hand, stiff aorta is the main cause of generating the higher blood pressure, particularly in mean arterial pressure and pulse pressure. This concept is supported by research provided by Bachelet-Rousseau et al. [49]. The authors although, as mentioned above, did not show during the annual observation a significant improvement in the elastic properties of the arteries, found a significant decrease of both diastolic and mean pressure. This allowed the authors to put forward suggestion, that perhaps the longer observation of patients would bring an answer, how the improving indices directly reflect the severity of the phenomenon of arterial stiffness.

Based on currently obtained results and approachable published data, it can be stated that the RT is a highly effective method of treatment, which allows to reverse the adverse structural and functional changes associated with ESRD and renal replacement therapy.

### Limitations of the study

We accept that this study has some limitations. This is a small-scale study using current echocardiographic methodology. The study group is not very large. This is due to the fact that the kidney transplants are relatively rare in Poland. In addition, the study included only patients who remained in full observation, and in whom all echocardiographic measurements was done. Another limitation is that, causes of kidney failure may affect remodeling of the aorta. Patients recruited to this study were being assessed for kidney transplantation and may not be representative of all patients with ESRD. We believe these results would be relevant to other patients with more significant comorbid conditions. Use of ACE inhibitors, statin use, antihypertensives and immunosuppression may have impact on vascular parameters. Other factors including duration of dialysis prior to renal transplant and biochemical markers can also modify the elastic properties of the aorta. The impact of the causes of ESRD and laboratory parameters on echocardiographic indices were not analyzed for two reasons. Firstly, the group was too small to analyze the impact of the etiology on echocardiographic parameters (eg. there was only 1 patient with chronic tubulo-interstitial nephritis, and in 4 cases the etiology was not known). Statistical analysis with such a small number of patients in the subgroups does not make sense. Second, study was strictly echocardiographic, what is highlighted in the title.

## Conclusions

There is a relationship between renal transplantation and improvement of the aortic elastic properties. Withdrawal of renal insufficiency allows to initiate the reparative processes leading to at least partial restitution of structure and function of the aorta, which is called reverse remodelling. Improvement of aortic elastic properties after renal transplantation is a continuous and prolonged process.

## Abbreviations

ACE: Angiotensin converting enzyme
AD: Aortic distensibility
ADMA: Asymmetric dimethylarginine
Ao_max_: Aortic maximal diameter
Ao_min_: Aortic minimal diameter
AS: Aortic strain
ASI: Aortic stiffness index
BMI: Body mass index
BSA: Body surface area
CAD: Coronary artery disease
CI: Cardiac index
CO: Cardiac output
DBP: Diastolic blood pressure
EF: Left ventricle ejection fraction
ESRD: End-stage renal disease
FS: Left ventricle shortening fraction
HR: Heart rate
IVSDd: Interventricular septum diastolic diameter
IVSSd: Interventricular septum systolic diameter
LV: Left ventricle
LVEDd: Left ventricle enddiastolic diameter
LVEDV: Left ventricle enddiastolic volume
LVESd: Left ventricle endsystolic diameter
LVESV: Left ventricle endsystolic volume
LVH: Left ventricle hypertrophy
LVM: Left ventricle mass
LVMI: Left ventricle mass index
MBP: Mean blood pressure
NOS: Nitric oxide synthase
PP: Puls pressure
PWDd: Posterior wall diastolic diameter
PWSd: Posterior wall systolic diameter
RT: Renal transplantation
SBP: Systolic blood pressure
SI: Stroke index
SV: Left ventricle stroke volume
TTE: Transthoracical echocardiography
